# The role of TDP-43 mislocalization in amyotrophic lateral sclerosis

**DOI:** 10.1186/s13024-020-00397-1

**Published:** 2020-08-15

**Authors:** Terry R. Suk, Maxime W. C. Rousseaux

**Affiliations:** 1 University of Ottawa Brain and Mind Research Institute, Ottawa, Canada; 2 Department of Cellular and Molecular Medicine, University of Ottawa, Ottawa, Canada; 3 Eric Poulin Center for Neuromuscular Diseases, Ottawa, Canada; 4 Ottawa Institute of Systems Biology, Ottawa, Canada

**Keywords:** ALS, TDP-43, Mislocalization, Pathology, Nucleocytoplasmic shuttling

## Abstract

Since its discovery as a primary component in cytoplasmic aggregates in post-mortem tissue of patients with Amyotrophic Lateral Sclerosis (ALS), TAR DNA Binding Protein 43 kDa (TDP-43) has remained a central focus to understand the disease. TDP-43 links both familial and sporadic forms of ALS as mutations are causative for disease and cytoplasmic aggregates are a hallmark of nearly all cases, regardless of TDP-43 mutational status. Research has focused on the formation and consequences of cytosolic protein aggregates as drivers of ALS pathology through both gain- and loss-of-function mechanisms. Not only does aggregation sequester the normal function of TDP-43, but these aggregates also actively block normal cellular processes inevitably leading to cellular demise in a short time span. Although there may be some benefit to therapeutically targeting TDP-43 aggregation, this step may be too late in disease development to have substantial therapeutic benefit. However, TDP-43 pathology appears to be tightly linked with its mislocalization from the nucleus to the cytoplasm, making it difficult to decouple the consequences of nuclear-to-cytoplasmic mislocalization from protein aggregation. Studies focusing on the effects of TDP-43 mislocalization have demonstrated both gain- and loss-of-function consequences including altered splicing regulation, over responsiveness to cellular stressors, increases in DNA damage, and transcriptome-wide changes. Additionally, mutations in *TARDBP* confer a baseline increase in cytoplasmic TDP-43 thus suggesting that small changes in the subcellular localization of TDP-43 could in fact drive early pathology. In this review, we bring forth the theme of protein mislocalization as a key mechanism underlying ALS, by highlighting the importance of maintaining subcellular proteostasis along with the gain- and loss-of-functional consequences when TDP-43 localization is dysregulated. Additional research, focusing on early events in TDP-43 pathogenesis (i.e. to the protein mislocalization stage) will provide insight into disease mechanisms, therapeutic targets, and novel biomarkers for ALS.

## Background

### TDP-43, a central player in amyotrophic lateral sclerosis

Amyotrophic lateral sclerosis (ALS) is a fatal neurodegenerative disease characterized by the selective loss of motor neurons resulting in mortality within an average of 2-5 years [1]. Though most cases of ALS are sporadic (sALS), approximately 10% are familial (fALS) in origin. The identification of these familial cases, now spanning over 20 genes (reviewed by Nguyen et al. [2]) has highlighted the importance of various cellular processes in the pathogenesis of ALS [3]. Indeed, some rare genetic cases – such as the identification of mutations in *TAR DNA Binding Protein 43 kDa (TARDBP*, encoding TDP-43) have provided crucial insight into common pathogenic themes in ALS [4–7].

TDP-43 bridges the divide between sporadic and familial ALS and remains a dominant protein of interest to understand disease pathogenesis. TDP-43 was identified as a primary component of ubiquitinated and hyper-phosphorylated cytosolic aggregates observed from post-mortem tissue of patients with ALS [8, 9]. This pathological phenomena is considered a hallmark of ALS as it is observed in approximately 97% of all ALS patients regardless of the mechanisms of disease onset, with the notable exceptions of familial ALS (fALS) caused by mutations in *Zn/Cu Superoxide Dismutase 1* (*SOD1*) and *Fused in Sarcoma* (*FUS*) [1, 10–15]. Furthermore, since the first report in 2008, over 50 mutations in *TARDBP* have been linked to ALS, further supporting TDP-43 dysfunction as a critical component in ALS [4–6, 16–18]. Therefore, TDP-43 dysfunction provides common ground in an otherwise convoluted disease, thus gaining notoriety and attention from researchers aiming to uncover the mechanisms causing TDP-43 aggregation. It is also important to note that mutations in *TARDBP* can also cause frontotemporal lobar dementia (FTLD), which itself shares some clinical parallels with ALS and displays TDP-43 pathology in ~ 45% of cases [8, 9, 19–21]. Here, however, we will focus on TDP-43 dysfunction as a central mechanism connecting multiple pathways in the context of ALS.

## Main text

### TDP-43 function, dysfunction, and aggregation

TDP-43 is a highly conserved and essential DNA/RNA binding protein belonging to the heterogenous ribonucleoprotein family that preferentially recognizes UG-rich and TG-rich motifs of RNA and DNA, respectively [22–26]. TDP-43 is ubiquitously expressed in all cell types and is predominantly localized to the nucleus, but is also present in the cytoplasm and mitochondria [27–29]. Importantly, TDP-43 is highly regulated, particularly by autoregulation through cryptic exon repression within the 3’UTR of *TARDBP* mRNA [30–32]. Deletion of TDP-43 results in embryonically lethality in mice, and its depletion or overexpression causes toxicity or cell death in cell and animal models [33–48]. Structurally, TDP-43 has a bipartite NLS sequence in the N-terminal domain upstream of the first RNA recognition motif (RRM), a nuclear export signal (NES) within the second RRM, and 5 putative mitochondria localization signals (M1-M5) of which 3 (M1, M3, and M5) are functionally characterized [14, 24, 28, 29]. The NLS and NES are important for shuttling TDP-43 between the nucleus and cytoplasm, however the involvement of the NES remains controversial as some studies suggest the NES is non-functional [27, 49–51]. These motifs reside within the N-terminal portion of TDP-43 forming a globular tertiary structure [22, 52, 53]. The C-terminal domain (CTD) – sometimes referred to as the low-complexity domain (LCD), glycine-rich region, intrinsically disordered region (IDR), or prion-like domain (PrLD) – remains relatively unstructured and is thought to be critically important for TDP-43 toxicity in disease [4, 53, 54]. Not only is the unstructured nature of the CTD aggregation-prone, but nearly all ALS-causing mutations on TDP-43 cluster within this domain [4, 6, 7].

**Fig. 1:**
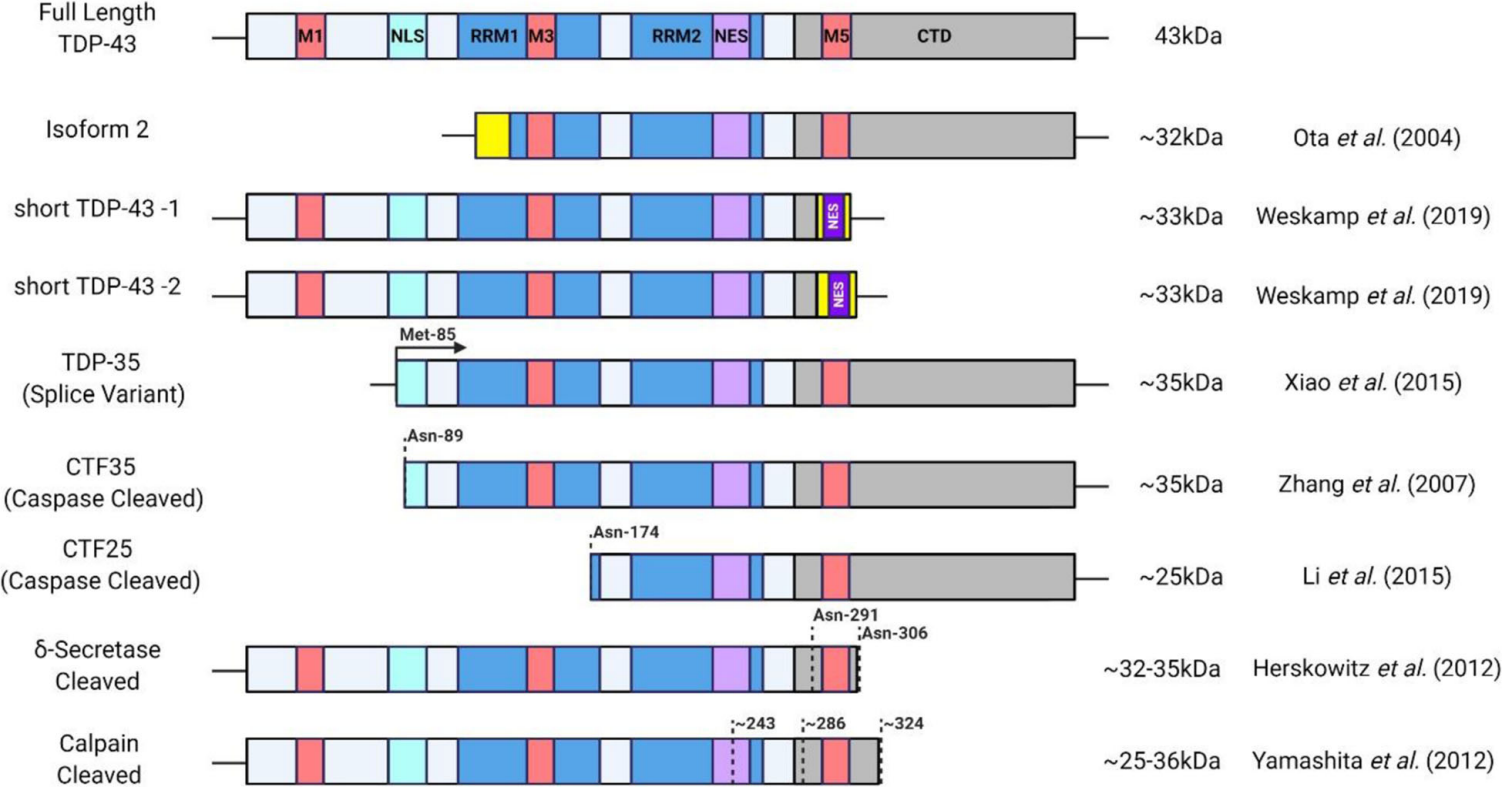
Structure of TDP-43 including functional domains and identified short-species. M1,M3,M5 (Red): Mitochondria Localization Sequences; NLS (Turquoise): Bipartite Nuclear Localization Sequence; RRM1,RRM2 (Blue): RNA Recognition Motif; NES (Light Purple): Controversial Nuclear Export Signal; NES (Dark Purple): Nuclear Export Signal; CTD (Grey): C-Terminal Domain; Yellow Box: Alternate Amino Acid Sequence (N-Terminus of “Isoform 2” and C-Terminus of “Short TDP-43”); Dashed Lines: Cleavage Sites

In ALS, truncated forms of TDP-43 are found in ALS aggregates, more predominantly in the cortex but also to a lesser extent in the spinal cord [55–59]. The N-terminally truncated, C-terminal fragments 35 kDa (CTF35) and 25 kDa (CTF25) are the most notable “species” of TDP-43 [8, 60–62]. Several species of TDP-43 exist and are produced through translation of alternatively spliced isoforms or through proteolytic cleavage at the post-translational level (Fig. 1). CTF35 and CTF25 can be generated through proteolytic cleavage via Caspases 3 and 7 after asparagine-89, and Caspase 4 after asparagine-174, respectively, and caspase activity is also modulated by the ALS-linked protein Progranulin (PGRN) [63–69]. Alternative splicing also contributes to short forms of TDP-43 where a second splice isoform was identified through cDNA sequencing encoding an N-Terminally truncated, ~ 32 kDa isoform of TDP-43 [70]. Additionally, CTF35 fragment can also be generated through non-canonical splicing in exon 2 and alternative translational initiation at methionine-85 [59]. C-terminally truncated species can also be generated through proteolytic cleavage. δ-secretase cleaves TDP-43 after asparagine-291 and -306 to generate a ~ 32 kDa and ~ 35 kDa species, respectively [71]. The calcium-dependant cysteine proteases, calpains, also play a role in TDP-43 cleavage generating ~ 35 kDa and ~ 25 kDa species associated with cell toxicity [72, 73]. As many of the truncated species of TDP-43 are of similar molecular weights many studies simply nest them as “CTF35” or “CTF25” based on molecular weight without investigation to the exact species observed which may limit the understanding of TDP-43 species contribution to ALS as different species display distinctive properties [59, 63, 74, 75]. The exact functions of these truncated species remain unclear and are generally thought to be toxic, but have also been proposed to serve a protective role in the cell to promote TDP-43 clearance [59, 63, 73, 75–80]. It is important to recognize that other species of TDP-43 CTFs have been identified at 15-16 kDa, 22-25 kDa, and 33-37 kDa in ALS/ALS-FTLD, however due to low levels of reporting their prevalence in disease remains elusive [56, 74, 75, 81–85].

There are several features that commonly define aggregates of TDP-43 in ALS. These include the accumulation of post translational modifications such as ubiquitination, poly-ubiquitination, and aberrant phosphorylation (sometimes referred to as hyperphosphorylation) of full length TDP-43; specifically phosphorylation of TDP-43 at serine 409 and 410 (S409/410) is widely used as an indicator of aggregated TDP-43 [8, 9, 56, 58, 61]. TDP-43 aggregates in ALS also accumulate full length and lower molecular weight species of TDP-43 and stain positive for the ALS-linked ubiquitin-binding autophagic adaptor Sequestosome 1 (SQSTM1, also known as p62) [8, 55–60, 82, 86–89].

The exact mechanisms mediating the formation of TDP-43 aggregates remain elusive. In ALS aggregates, TDP-43 was found to colocalize with important markers of stress granules (SGs) [90–95]. SGs are membraneless organelles that form in the cytoplasm comprised primarily of ribonuclear proteins and mRNA stalled in translation (Reviewed by Wolozin & Ivanov [96]). The formation of SGs occurs through a process called liquid-liquid phase separation (LLPS) where SG proteins and associated mRNA will de-mix into a liquid phase distinct from the cytosol [97, 98]. Two prominent proteins that are indicative of a SG are Ras GTPase-activating protein-binding protein 1 (G3BP1) and TIA1 cytotoxic granule-associated RNA binding protein (TIA1, [99–102]). Interestingly, mutations in the LCD of TIA1 – a domain that plays a key role in LLPS – cause ALS, further supporting the involvement of the cellular stress response in disease [103].

TDP-43 plays an important role in regulating the dynamics of SG formation and disassembly where loss of TDP-43 reduces SG formation [104, 105]. Treatment of cells with cell stressors used to study the formation of SGs, such as oxidative stressors (e.g. Sodium Arsenite), osmotic stressors (e.g. D-Sorbitol) or heat shock, results in the formation of phase-separated TDP-43 structures in the cytoplasm. Nevertheless, there remains a debate in the field as to whether cytoplasmic TDP-43 structures indeed colocalize as a component of SGs or are mostly distinct from these bodies [90, 91, 93, 94, 103, 104, 106–109]. Under prolonged stress conditions, phase-separated TDP-43 transitions from a liquid-like droplet to form gel-like inclusions inhibiting their ability to dissociate [110–112]. These gel-like inclusions eventually accumulate several hallmarks the TDP-43 inclusions seen in ALS [61, 108, 109, 111].

Clearance of TDP-43 remains an important biological process tightly coupled with cytotoxicity. The ubiquitin-proteasome system is disrupted by ALS-linked mutations in *Ubiquilin-2* (*UBQLN2*), and is important for degrading full-length TDP-43 in addition to CTF-35 and CTF-25 [113–120]. Inhibiting this mode of clearance in primary neurons results in a greater accumulation of cytoplasmic TDP-43 aggregates compared to other cell stressors [113, 121, 122]. Recently a gain-of-function mutation in *CYLD Lysine 63 Deubiquitinase* (*CYLD*) was identified to cause ALS and FTLD [123]. The authors demonstrated in mouse primary neurons that this mutation increased deubiquitinase activity, decreased autophagy function and caused TDP-43 mislocalization, along with TDP-43 aggregation in the human brain. Autophagy also plays a role in clearing aggregated forms of TDP-43 and is linked to ALS through mutations in autophagy-related proteins *SQSTM1*, *TANK Binding Kinase 1* (*TBK1*) and *Optineurin* (*OPTN*) [87, 113, 124–128]. Of particular importance, the sequestration of SQTSM1 into TDP-43 aggregates, one of the aforementioned hallmarks of ALS aggregates, leads to the inhibition of proteasome function in addition to autophagy, further promoting the accumulation of toxic, misfolded proteins in cells [129, 130].

The reduced clearance of aggregates can lead to another toxic gain-of-function mechanism: blocking intracellular transport. Aggregates are observed throughout the cytoplasm, often in the soma, but are also observed in the axons and dendrites [131, 132]. Inhibiting axonal transport is a common feature in ALS and particularly relevant as mutations in genes involved in cellular transport, namely *KIF5A* or *DCTN1*, cause ALS [133–139]. This may provide some insight into selective neuron vulnerability in ALS as motor neuron axons are particularly long and susceptible to changes in trafficking dynamics [139]. Additionally, TDP-43 plays an important role in axonal trafficking of mRNA granules, a function lost when it is mutated or aggregated [131, 132, 140–142].

### Additional avenues of TDP-43 toxicity

Studies have suggested that not all aggregates are equal in their ability to cause toxicity. Similar to other neurodegenerative diseases, large protein aggregates such as amyloid-like structures may not be as toxic as smaller ones that preceded them such as oligomers [143–147]. However, describing aggregates simply as “large” or “small” is a gross oversimplification as there are thought to be multiple species of aggregates based on the properties of protein misfolding which may mediate altered toxicity at different stages [53, 147–150]. Although TDP-43 aggregation is apparent in various modes of cellular dysfunction, critics argue that TDP-43 aggregates may simply be an artifact of neuronal degeneration observed at the time of post-mortem analyses [14, 151]. In cell and animal models, TDP-43 aggregation is not necessarily essential to cause cellular toxicity [14, 152–157]. This would suggest that TDP-43 aggregates may act as a bystander alongside a cell death pathway or work in parallel with an alternate mechanism to promote toxicity. Furthermore, TDP-43 aggregates are not exclusive to motor neurons, they can also be observed in glia and muscle tissue of ALS patients and are observed to spread in a prion-like manner throughout the brain [8, 9, 15, 57, 158–162]. Yet in ALS, motor neurons selectively degenerate suggesting that the presence of TDP-43 aggregates may not necessarily *drive* cell-death. Clearly, TDP-43 aggregation is not the only feature at play.

In the presence of ALS-causing mutations, TDP-43 often demonstrates an altered nucleocytoplasmic distribution (increased cytosolic, decreased nuclear) in comparison to its wild-type counterpart [153, 163]. This may suggest that TDP-43 dysfunction can promote cytoplasmic accumulation. However, it remains difficult to differentiate whether cellular phenotypes may be caused by mislocalization or the mutation itself. Studies have exploited the NLS sequence on TDP-43 through genetic manipulation to shed light on the consequences of mislocalization independent of mutations or aggregate formation as in the cell stress models. In cellular models, expression of TDP-43^ΔNLS^ resulted in depletion of endogenous TDP-43^WT^ from the nucleus and promoted the formation of insoluble inclusions in the cytoplasm [29]. In a transgenic mouse model expressing human TDP-43^ΔNLS^ under a neurofilament heavy chain promoter for brain and spinal cord expression, mice displayed a rapidly progressive motor phenotype, loss of body mass, neuromuscular denervation, and spinal motor neuron loss [155]. These mice also exhibited high levels of phosphorylated TDP-43 aggregates throughout the brain and spinal cord. Interestingly, the authors of this study describe progressive endogenous nuclear TDP-43 depletion followed by aggregate formation in the brain and to a lesser extent the spinal cord. This infers that TDP-43 mislocalization can promote nuclear depletion and is likely upstream of aggregation. Much of the toxicity, however, was attributed to the high level of transgene expression in the animal model which can function to exacerbate the effect of induced TDP-43 mislocalization by inducing cellular stress from TDP-43 overexpression.

**Fig. 2:**
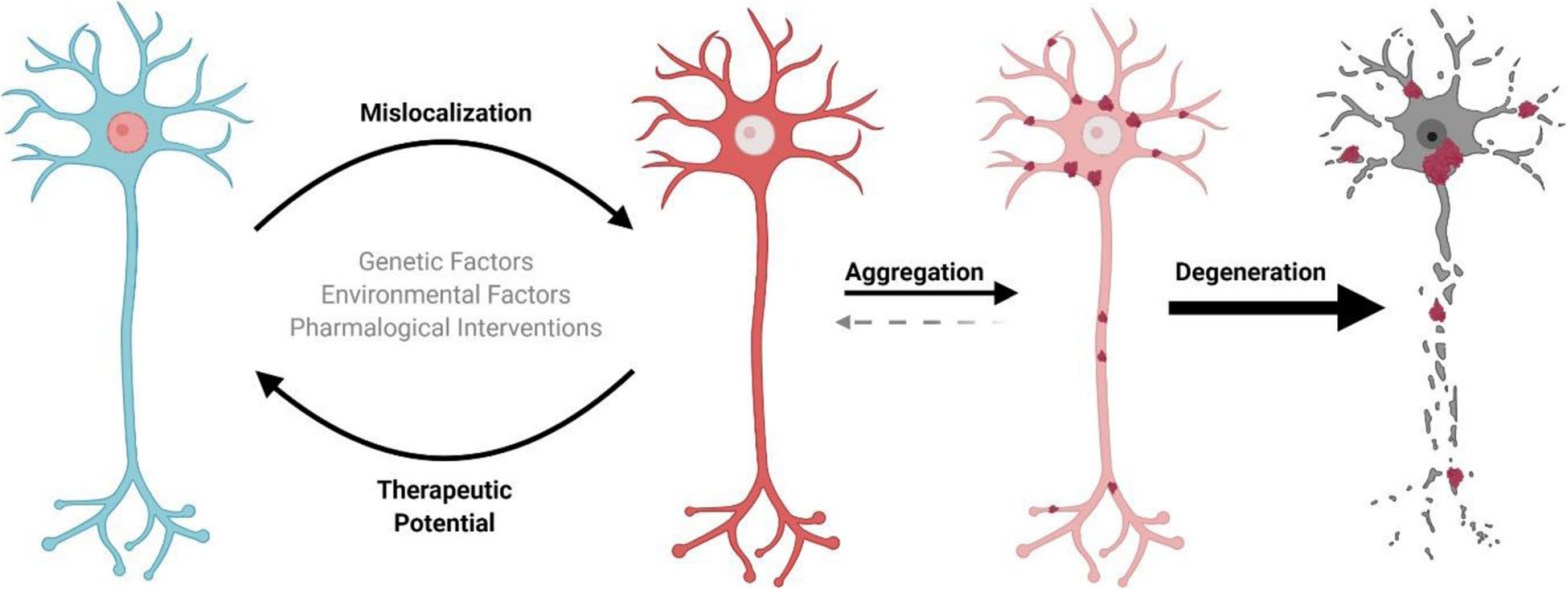
TDP-43 (Red) mislocalizes (partially or completely) from the nucleus to the cytoplasm due to genetic and/or environmental factors causing deleterious effects to the cell. Prolonged mislocalization promotes aggregation. Under physiological conditions the cell can clear small TDP-43 aggregates through proteasomal, endosomal, or autophagic degradation. Prolonged The accumulation of TDP-43 aggregates disrupts physiological functioning (e.g. sequestration of SQSTM1) thereby exacerbating pathology and promoting neuronal degeneration. Early interventions normalizing TDP-43 localization hold the potential to prevent cellular demise

The cellular stress model, particularly oxidative stress (e.g. via sodium arsenite treatment), to induce SGs and TDP-43 inclusions is a widely used model to study TDP-43 mislocalization and aggregation [96, 164]. Yet systemic stress makes it difficult to differentiate phenotypes associated with TDP-43 mislocalization and accumulation to general cellular stress responses. To overcome this limitation, a novel model expressed TDP-43 fused with an *Arabidopsis thaliana-*derived Cryptochrome 2 (CRY2) protein to allow for optogenetic instigation of LLPS [108, 109, 111, 165]. In contrast to prolonged sodium arsenite treatment, prolonged LLPS through optogenetic stimulation in wild-type conditions results in TDP-43 inclusions within the nucleus absent of ALS hallmarks including hyperphosphorylation and SQSTM1 sequestration [108]. However, prolonged induction of LLPS on TDP-43^ΔNLS^ or mildly stressed cells inducing a mild mislocalization resulted in cytoplasmic inclusions positive for ALS-like hallmarks. Additionally this model demonstrated in vitro that there is a relatively short time course between initial induction of LLPS to aggregate formation (within hours) and inevitable cell death in less than 6 weeks [108, 111]. This suggests a slippery slope between aggregate formation and neurodegeneration, thereby inferring that therapeutically targeting the aggregation step may be too late to have substantial impact on disease progression (Fig. 2). Increasingly, the field is focusing on mechanisms outside of TDP-43 aggregation to identify early drivers of disease.

### The contribution of TDP-43 mislocalization to cellular toxicity in ALS

Increasing evidence suggests that nuclear-to-cytoplasmic mislocalization of TDP-43 induces toxicity through both loss- and gain-of-function mechanisms. Classic roles for TDP-43 pertain to mRNA maturation in the nucleus, specifically acting as a repressor of alternate splicing, cryptic exon splicing, and alternate polyadenylation [25, 166–173]. Loss of these functions through mislocalization or depletion have widespread deleterious effects on the cell [170, 173]. For example, recently it was discovered that loss-of-TDP-43 decreases microtubule outgrowth specifically in motor neurons through premature polyadenylation of the Stathmin2 (*STMN2*) transcript [167, 174]. TDP-43 is also involved in mRNA transport, a mechanism that is dysregulated within ALS, as well as local translational regulation [131, 175]. Disruption of either of these mechanisms may effectively trap TDP-43 in the cytoplasm, inhibiting its normal functions. This hypothesis is substantiated by transcriptomic evidence showing that diseased neurons and mouse models of ALS demonstrate increases in alternative splicing events, cryptic exon inclusion and alternate polyadenylated sequences [168, 176–178]. Recently, the CTD of TDP-43 was found to mediate its recognition of G-quadruplex structures on RNA, facilitating subcellular transport to neurites for local translation and nucleocytoplasmic trafficking [179, 180]. Interestingly, C9ORF72 hexanucleotide repeat expansion results in G-quadruplex formation, however the relationship between TDP-43 and C9ORF72 in the context of these structures has not yet been explored [181–183]. Additionally, RNA binding abilities of TDP-43 have been linked to TDP-43 toxicity, though some studies suggest that RNA binding is a protective mechanism [111, 184–189]. An important finding suggests that TDP-43 RNA binding regulates its solubility and lack of RNA promotes aberrant inclusions in the cytoplasm [111, 189]. Mislocalization of TDP-43 may inhibit proper RNA trafficking to the cytoplasm and subsequently promote an environment where TDP-43 is less soluble.

The nuclear functions of TDP-43 are not limited to its RNA binding functions; TDP-43 also binds DNA at TG-rich regions to regulate gene expression and exon skipping [25, 190]. For example, TDP-43 normally binds to the promoter of *Vacuolar Protein Sorting 4B* (*VPS4B*) to repress its transcription [191]. Loss of function due to mislocalization results in a loss of *VPS4B* repression leading to an increased interaction with the ALS-linked protein Charged Multivesicular Body Protein 2B (CHMP2B) thereby disrupting dendritic recycling-endosome trafficking and reducing ALS-linked ERB-B2 Receptor Tyrosine Kinase 4 (ERBB4) surface expression [191–193]. Another nuclear role for TDP-43 is in its response to genomic double stranded breaks (DSBs) which accumulate in ALS patients [163, 194–200]. Mislocalization of TDP-43 through an ALS-causing mutation impair the nuclear localization of DSB-repair proteins and result in the accumulation of DNA damage promoting cell death [163, 194, 201, 202]. Loss of nuclear TDP-43 can also affect chromatin accessibility leading to altered gene expression [203, 204].

Not all consequences of TDP-43 mislocalization are attributed to nuclear loss-of-function as TDP-43 has defined roles in the cytoplasm including stress granule regulation, mRNA stability, translational regulation, local synaptic RNA regulation, mRNA trafficking, microRNA regulation, and regulation of autophagy (extensively reviewed by Birsa et al. [205]). The exact consequences of increased cytoplasmic TDP-43 on these cellular functions remains largely unknown as most studies focus on protein aggregation resulting in an effective loss-of-TDP-43 function. TDP-43 is cleared through both the ubiquitin-proteasome system and lysosomal degradation pathways (highlighted above) [206–208]. Interestingly, TDP-43 mislocalization through overexpression or pathogenic mutations causes vacuole fragmentation, causing cellular disruption in addition to altering its own clearance [206]. Additionally, mislocalization may prime cells to respond abnormally in certain circumstances. As TDP-43 can readily undergo phase separation, the increase in cytoplasmic density biophysically promotes LLPS to occur (reviewed by Boeynaems et al. [98]). This is apparent in models of cellular stress when TDP-43 is mislocalized as there is a significant increase in the cellular stress response including rapid formation of stress granules and TDP-43 granules [109, 111, 165]. Therefore, mislocalization may sensitize the cell to respond disproportionately to a cellular stress than it may normally be less responsive to. This was recently exemplified in a study where induced pluripotent stem cell (iPSC)-derived motor neurons, but not astrocytes, with mislocalized TDP-43 showed an increase level of cell death when seeded with TDP-43 aggregates from patient tissue [209].

Although we have focused strictly on nuclear and cytoplasmic TDP-43, it is important to highlight a role for TDP-43 at mitochondria. TDP-43 misregulation through genetic manipulation of the NLS, presence of an ALS-causing mutation, or overexpression result in an increased localization to mitochondria [28]. Within the mitochondrion, mutant TDP-43 also preferentially binds mitochondria-resident mRNA, presumably causing Complex 1 disassembly through altered expression of its components [28]. Nevertheless, this finding has been debated, as studies in cell and animal models of ALS suggest that mitochondrial energetics and metabolism are unaltered [210]. The important contribution of mitochondria in ALS however remains a focus due to mutations in the mitochondrial protein SOD1 as a primary genetic cause of fALS [11]. However, it is interesting to consider that fALS caused by mutations in *SOD1* rarely present with TDP-43 pathology [15].

Together, these data suggest that both loss- and gain-of-TDP-43 function mediated by nuclear-to-cytoplasmic mislocalization cause systemic cellular dysfunction in ALS. This recognition calls for a better understanding of the native subcellular functions of TDP-43 and the consequences of mislocalization independent of aggregation.

### Potential mechanisms driving TDP-43 Mislocalization

It is apparent that TDP-43 mislocalization on its own is toxic and can contribute to many of the cellular characteristics observed in ALS. However, the mechanisms governing TDP-43 localization remain largely elusive. Identifying the mechanisms driving mislocalization will be crucial to identify key mechanisms that are misregulated early in disease and can be therapeutically targeted to prevent TDP-43 pathology all together (Fig. 2). These mechanisms can cover a range of biological aspects intrinsic to TDP-43 function as well as systemic cellular function [202].

As previously described, TDP-43 contains several subcellular-regulatory sequences including an NLS, the controversial NES, and mitochondrial localization sequences M1, M3, and M5. ALS-causing mutations however rarely reside within these motifs (with the exceptions of A90V in the NLS, and mutations between amino acids 294-300 in M5), and TDP-43 mislocalization exists outside of *TARDBP* mutations, suggesting extrinsic factors from TDP-43 govern its subcellular localization [4, 6, 28, 151, 211–222]. Understanding the contribution of these domains to TDP-43 biology remains an important step to understand disease. To this end, targeted mutagenesis of TDP-43 NLS sequence suggests that TDP-43 is actively transported into the nucleus [49]. Furthermore, knockdown of nuclear import machinery (e.g. Importin-β) impair TDP-43 nuclear localization, increasing the cytoplasmic abundance of TDP-43 [223]. Mutagenesis of the NES does not alter TDP-43 localization suggesting the NES is non-functional, however manipulation of export machinery (e.g. Exportin 1) yields conflicting results; thus there may be overlapping mechanisms of TDP-43 export [49–51]. Nevertheless, nuclear pore trafficking is important to some extent for normal TDP-43 localization. Perhaps unsurprisingly, in ALS, nuclear pore trafficking is disrupted, especially in cases of patients bearing mutations in *TARDBP* or *C9ORF72* [223–228]. Aggregates of TDP-43 sequester nuclear pore proteins which would likely exacerbate TDP-43 mislocalization and accumulation into the protein aggregates [224]. In cases of *C9ORF72* repeat expansion, dipeptide repeats (DPRs) generated through repeat-associated non-AUG (RAN) translation of the expanded hexanucleotide (CCCCGG) repeat blocks and disrupts the nuclear pores leading to TDP-43 pathology [229–235]. TDP-43 mislocalization was also shown to exacerbate RAN translation of *C9ORF72* DPRs and could contribute to nuclear pore defects in conjunction or independently from DPRs [235]. This study suggests that C9ORF72 neurotoxicity may be mediated by TDP-43, and that TDP-43 mislocalization independent of *C9ORF72* DPRs can disrupt nuclear pore function. Thus, nuclear pore complex disruption is an important part of TDP-43 pathogenesis, however, this mechanism may not always precede TDP-43 mislocalization. These studies warrant further investigation into mechanisms that may hinder TDP-43 translocation into the nucleus as potential aggravators of disease.

It is clear that regulation of TDP-43 is crucial for proper function, yet relatively little is known about how TDP-43 is regulated. Post translational modifications play an important role in regulating protein function (Reviewed by Buratti [236]). Along with phosphorylation at S409/410, toxic TDP-43 generally displays an overabundance of phosphate modifications leading to the general consensus that phosphorylation of TDP-43 is toxic [58, 61, 237–245]. However, phosphorylation may play a protective role and promote normal function within the cell [246]. For example, phosphorylation of TDP-43 at T153 and T155 by Mitogen Activated Protein Kinase Kinase (MEK) regulates TDP-43 localization to the nucleolus after heat shock, suggesting a normal maintenance role for phosphorylation in TDP-43 biology [247]. Roles for other post translational modifications such as acetylation, poly-ADP ribosylation (PARylation), oxidation, and ubiquitination have been described suggesting that post translational modifications are likely important for normal TDP-43 function and may have unappreciated roles regulating subcellular localization [112, 236, 248–250].

The role of TDP-43 cleavage into CTF35 and CTF25 is gaining traction as potential contributors of normal and toxic TDP-43 function. CTF35 for example assembles into SGs and plays roles in RNA processing, however, CTF25 does not localize to SGs and remains diffuse throughout the cell [251]. Cytoplasmic localization of CTF35 and CTF25 may be due to the partial and full loss of the bipartite NLS upon cleavage, respectively [59, 63, 252]. Additionally, mutations in TDP-43 and CTF35 also increase mitochondrial localization to the mitochondrial matrix and intermembrane space, respectively [252]. This study highlights that full length TDP-43, not CTF35, may cause oxidative stress, in turn increasing TDP-43 cleavage and promoting mislocalization and aggregation. As these fragments are observed in TDP-43 aggregates, increased in disease, and may induce neuronal toxicity, they remain an interesting mechanism that may provide insight into TDP-43 biology and ALS [64, 77, 251–253]. Several studies have identified alternative spliced isoforms of TDP-43, yet few have been functionally characterized [22, 30, 254]. Recently, a C-terminally truncated alternatively spliced isoform of TDP-43 (“short TDP-43” or sTDP-43) was characterized and found to encode a functional NES within the alternative C-terminus (Fig. 1) resulting in a more cytoplasmic localization compared to TDP-43 [255]. Interestingly, sTDP-43 was upregulated in response to increased neuronal activity, induced mislocalization of endogenous TDP-43, and caused neurotoxicity. Additionally, the sTDP-43 isoform was abundant in TDP-43 aggregates from ALS patients’ spinal cord and tissue samples. Clearly, focusing exclusively on full length TDP-43 is not encompassing to understand its contribution to ALS. Further understanding the biological roles and consequences of cleaved and alternately spliced forms of TDP-43 will provide novel insight into ALS pathogenesis and aid our interpretations of TDP-43 contributions to disease.

### Approaches to study TDP-43 Mislocalization to better understand ALS

As with TDP-43 aggregates, interpreting the extent of TDP-43 mislocalization in patient tissue remains challenging as observations are made post-mortem at the late stage of disease. It may be implied that mislocalization of TDP-43 has occurred where there are cytoplasmic TDP-43 aggregates, however the mechanism(s) of biogenesis of this pathogenic hallmark remain(s) elusive. Future studies should systematically analyze the extent of TDP-43 mislocalization in addition to aggregation in human tissue to gain a better understanding of TDP-43 pathogenesis. Relying on key late-stage hallmarks such as phosphorylation of TDP-43 or SQSTM1 sequestration may limit the understanding of early pathogenesis and may lead to the dismissal of models that do not recapitulate late-stage pathology. Though studies have not systematically analyzed the extent of TDP-43 mislocalization throughout the central nervous system, data suggest that TDP-43 mislocalization correlates with aging in the vulnerable motor neurons of mouse spinal cord tissue [256]. Further understanding the basic biology and general extent of TDP-43 mislocalization throughout the central nervous system will help gain insight into the cell-type specific vulnerabilities to key stages of TDP-43 pathology.

There is a need for developing better models that recapitulate important aspects of ALS, including behavioural, pathological, and molecular phenotypes to further understanding of disease. Although most cases of ALS display TDP-43 proteinopathy, only some transgenic mouse models, and fewer endogenous mouse models, recapitulate TDP-43 pathology and ALS-like phenotypes (ALS mouse models recently reviewed by De Giorgio et al. [257]). An interesting exception is in mice bearing mutations in *Sen*a*taxin* (*SETX*), a poorly understood protein thought to act as a DNA/RNA helicase which cause a rare juvenile-onset form of fALS and sALS [258–260]. Both mouse models bearing transgenic and endogenous mutations in *SETX* recapitulate TDP-43 mislocalization, aggregation, and ALS-like phenotypes observed in patients [258]. Within *TARDBP* models, many models that display TDP-43 proteinopathy rely on overexpression approaches. With the advent of genome engineering (e.g. via CRISPR/Cas9) in animals and iPSCs, models have pushed towards studying endogenous TDP-43, moving away from the heavy reliance on transgenic and toxic overexpression models. Several knock-in mouse models expressing mutant TDP-43 display a range of phenotypes depending on the mutation, but most behavioural phenotypes are quite subtle and occur at a very-late stage [254, 257, 261, 262]. For example, the TDP-43^Q331K^ knock-in mouse model does not display significant motor phenotype whereas the TDP-43^Q331K^ transgenic mouse display some, but not robust, motor deficits [31, 263]. This difference may be due to synergistic effects of the TDP-43 mutation and overexpression in the transgenic model. There are several iPSC models for ALS (recently reviewed by Hawrot et al. [264]) however many of them do not recapitulate key hallmarks of ALS pathology. Neurons derived from TDP-43^A90V^ patient iPSCs display TDP-43 mislocalization (likely due to disruption of the NLS), TDP-43^M337V^ results in slight cytoplasmic granular staining of TDP-43 in iPSC-derived motor neurons, and mislocalization of TDP-43 is observed in TDP-43^M337V^ patient iPSC-derived astrocytes [265–267]. The difficulty in recapitulating TDP-43 pathology may suggest that there are additional mechanisms contributing to ALS in conjunction with TDP-43 mutations, such as changes associated with human aging or chronic stress on the cell. Attempts to exacerbate ALS pathology or behaviour deficits in physiologically relevant models may help to elucidate more complex mechanisms driving disease.

Although protein aggregation has gained the most attention to understand ALS and identify novel therapeutic targets, studying the earlier components of TDP-43 mislocalization may provide an important level of insight into the disease onset and progression. However, studying subcellular localization comes with its own challenges ranging from limitations in technology to convoluted interpretations. TDP-43 mislocalization is primarily studied using cellular fractionation methods or microscopy-based methods. Developing and optimizing more reliable methods to quantitatively analyze TDP-43 subcellular localization will enhance our understanding of critical regulators of TDP-43. For example, identifying nuclear-, mitochondrial-, and cytosol-specific post translational modifications will allow for the generation of antibodies facilitating more rapid and quantifiable detection of mislocalized TDP-43. These would parallel the antibodies raised against phospho-S409/410 TDP-43 which function as a gold-standard for detection of TDP-43 aggregates through microscopy [61]. The generation of reliable tools will help to resolve potential issues with subjectivity and enhance reproducibility to understand and characterize the consequences of TDP-43 mislocalization. Although much weight is placed on DNA sequencing for modern diagnosis, this technique offers relatively little diagnostic ability in the cases of complex diseases such as ALS. Additionally, DNA sequencing is generally limited to germline mutations and does not account for potential mosaicism which may arise during an individual’s lifetime which could lead to ALS [268]. For example, an ALS patient was reported to express TDP-43 bearing the ALS-causing Q331K mutation specifically in spinal cord neurons, but not in the occipital lobe suggesting this ALS-causing mutation is somatic and thus not likely identified through germline genome sequencing [163]. Biomarkers serve as a potential diagnostic feature in addition to providing insight into disease progression. Current biomarker candidates such as Neurofilament Light Chain are increased in ALS patients and may provide insight into disease progression, but is non-specific to ALS and only provides foresight by about 12 months before symptoms occur [269–273]. As TDP-43 mislocalization is a central feature in ALS and biomarkers based on phenotypes associated with mislocalization may provide the specificity and foreshadowing required for early diagnosis.

Integrative “omic” approaches will be important to identify robust biomarkers capable of diagnosis and providing early insight into disease progression. Determining the direct consequences of TDP-43 mislocalization as they pertain to ALS remains a challenge due to the incomplete understanding of TDP-43 function and general dysfunction associated with disease. Therefore unbiased, systems-based approaches will be important to understand TDP-43 biology surrounding mislocalization. Bulk RNA sequencing has provided great insight into transcriptomic changes in ALS mediated by TDP-43. This technology, however, has limited abilities to detect subtle biologically significant changes that may be cell-type specific. Incorporating single cell RNA sequencing or methods of enriching populations of interest (i.e. flow cytometry, spatial transcriptomics) will help target cell-type specific changes affected by TDP-43 mislocalization in animal and cell models that can translate to human disease [274, 275]. As TDP-43 plays important roles in regulating alternative splicing, alternate polyadenylation, and cryptic exon inclusion, deep RNA sequencing will help identify rare toxic species of RNA that can give insight into ALS progression or lead to biomarker for early disease [276, 277]. Interestingly, mislocalization of FUS was recently identified in iPSCs derived from patients bearing mutations in *Vasolin Containing Protein* (*VCP*) in addition to spinal cords from sALS patients [278]. FUS mislocalization was suggested to occur due to binding of aberrantly retained introns, namely in the *SFPQ* gene. The authors further suggest FUS mislocalization may be a more common hallmark of ALS than previously recognized however more evidence is required. Proteomic approaches such as immunoprecipitation-mass spectrometry or proximity-labeling mass spectrometry (e.g. APEX Proteomics or BioID) comparing wild-type to mislocalized TDP-43 will provide insight into locale-specific interactors [224, 279, 280]. Additionally, cross analysis with proteomic data from ALS tissue may provide insight into potential toxic protein-protein interactions as a result of TDP-43 mislocalization serving as early therapeutic targets. New technologies are also focusing on the subcellular localization of RNA such as APEXseq [281–283]. Enhancing this technology with datasets that monitor Protein-RNA binding (e.g. CLIP-seq), could greatly enhance our understanding of which transcripts are bound by TDP-43 in various subcellular compartments by comparing nuclear, cytoplasmic, and mitochondria TDP-43-RNA interactions [284]. Integrating these systems-based approaches will help to uncover novel markers of TDP-43 mislocalization and elucidate pathways leading to cellular demise in ALS.

## Conclusions

Increasing evidence suggests that TDP-43 aggregation is not a single driver of pathology in ALS. TDP-43 mislocalization plays significant roles in cellular dysfunction independently and in parallel to aggregation. Increasingly the field has begun to focus on understanding the regulatory mechanisms of TDP-43 mislocalization. To this end, as protein mislocalization is likely more readily reversible than protein aggregation, understanding the mechanisms regulating TDP-43 subcellular localization will be critical for therapy development. Specifically, a better understanding of TDP-43 localization regulators will surely shed light on novel therapeutics that have the potential to be more effective earlier in disease, more generalizable to *most* ALS cases, and more informative biomarkers for diagnosis and analysis of progression for ALS. Lastly, given that TDP-43 pathology can also coexist with other aggregate-prone proteins, such as C9ORF72 DPRs, Tau, α-Synuclein, and poly-Q expanded Huntingtin, insight into the role of TDP-43 mislocalization in its pathogenic function will serve to better understand pathology and modes of degeneration across a spectrum of neurodegenerative diseases [184, 285–288].

## Data Availability

Not applicable.

## Abbreviations

ALS: Amyotrophic Lateral Sclerosis
C9orf72: Chromosome 9 Open Reading Frame 72
CHMP2B: Charged Multivesicular Body Protein 2B
CNS: Central Nervous System
CRY2: Cryptochrome 2
CTD: C-Terminal Domain
CTF25: [TDP-43] C-Terminal Fragment 25 kDa
CTF35: [TDP-43] C-Terminal Fragment 35 kDa
CYLD: CYLD Lysine 63 Deubiquitinase
DCTN1: Dynactin 1
DSB: Double Stranded Breaks
ERBB4: RB-B2 Receptor Tyrosine Kinase 4 (ERBB4)
fALS: Familial ALS
FTLD: Frontotemporal Lobar Dementia/Frontotemporal Dementia
FUS: Fused in Sarcoma
G3BP1: Ras-GTPase Activating Protein-Binding Protein 1
iPSC: Induced Pluripotent Stem Cell
KIF5A: Kinesin Heavy Chain Isoform A
LCD: Low-Complexity Domain
LLPS: Liquid-Liquid Phase Separation
M1-M5: [TDP-43] Mitochondrial Localization Sequence 1-5
MEK: Mitogen Activated Protein Kinase Kinase
NES: Nuclear Export Signal
NLS: Nuclear Localization Signal
OPTN: Optineurin
PARylation: Poly-ADP Ribosylation
PGRN: Progranulin
PrLD: Prion-Like Domain
RAN: Repeat-Associated Non-AUG (translation)
RRM: RNA Recognition Motif
sALS: Sporadic ALSSG Stress Granule
SOD1: Zn/Cu Superoxide Dismutase 1
SFPQ: Splicing Factor Proline-, and Glutamine-Rich
SQSTM1: Sequestosome-1
sTDP-43: Short (Alternatively-Spliced) TDP-43
STXN: Senataxin
*TARDBP*: TAR DNA-Binding Protein 43 kDa (Gene)
TBC1D1: TBC1 Domain Family Member 1
TBK1: TANK Binding Kinase-1
TDP-43: TAR DNA-Binding Protein 43 kDa (Protein)
TIA1: TIA1 Cytotoxic Granule Associated RNA Binding Protein
UBQLN2: Ubiquilin-2
VCP: Vasolin-Containing Protein
VPS4B: Vacuolar Protein Sorting 4B
WT: Wild Type
